# Fiscal Policy for Health: Sugar-Sweetened Beverage Tax Design and Soft Drink Consumption Across Geographies, 2010–2024

**DOI:** 10.3390/ijerph23080985

**Published:** 2026-07-29

**Authors:** Rong Zheng, Hussain Ali, Yuxiao Hu, Mengmeng An

**Affiliations:** 1 WHO Collaborating Center on Health Tax and Fiscal Policy, School of International Trade and Economics (SITE), University of International Business and Economics (UIBE), Beijing 100029, China; rosezheng@uibe.edu.cn; 2 School of International Trade and Economics (SITE), University of International Business and Economics (UIBE), No. 10, Huixin Dongjie, Chaoyang District, Beijing 100029, China; hussainuibe@gmail.com (H.A.); 202400130013@uibe.edu.cn (Y.H.); 3 School of Government, University of International Business and Economics (UIBE), No. 10, Huixin Dongjie, Chaoyang District, Beijing 100029, China; 4 School of Law, University of International Business and Economics (UIBE), No. 10, Huixin Dongjie, Chaoyang District, Beijing 100029, China

**Keywords:** sugar-sweetened beverages, SSB taxation, fiscal policy for health, tax structures, health-oriented taxation, soft drink sales, difference-in-differences, non-communicable diseases

## Abstract

**Highlights:**

**Public health relevance—How does this work relate to a public health issue?**

- Sugar-sweetened beverages are an important source of added sugars and are linked to obesity, type 2 diabetes, cardiovascular disease, and other non-communicable diseases.
- This study examines SSB taxation as a fiscal policy tool for reducing per capita soft-drink sales volume across 74 geographies from 2010 to 2024.

**Public health significance—Why is this work of significance to public health?**

- SSB taxes were associated with a reduction of 7.286 L per person in soft-drink sales volume, equivalent to about 10.4% of the sample mean.
- The study contributes evidence not only on whether SSB taxes are associated with lower sales volume, but also on how tax-design features such as health objectives and tax structure may matter.

**Public health implications—What are the key implications or messages for practitioners, policy makers and/or researchers in public health?**

- Governments should consider SSB taxation as part of broader fiscal and nutrition policy packages for non-communicable disease prevention.
- SSB taxes may be more policy-relevant when they are clearly designed, communicated, and positioned as health-oriented fiscal measures.

**Abstract:**

Sugar sweetened beverages (SSBs) are an important source of added sugars and a key target for fiscal policies aimed at preventing obesity, type 2 diabetes and other non-communicable diseases. This study assessed the association of SSB taxes, tax structure and tax-design elements and changes in per capita soft drink sales volume across geographies from 2010 to 2024. Annual Euromonitor International Passport sales data were combined with national SSB tax-policy data from the World Bank and macroeconomic indicators. The final sample includes 74 geographies and 1096 geography-year observations. We use a multi-period difference-in-difference technique with staggered implementation of the policy and event-study estimation. SSB taxes were associated with a reduction of 7.286 L per person (*p* < 0.01), about 10.4% of the sample mean. Taxes with an explicit health objective were associated with larger reductions than taxes without a health objective. Ad valorem, mixed, and specific taxes were related with lower sales volume, although differences across structures were not significant. Importantly, the results imply policy design matters. This indicates that governments should evaluate not only whether to impose SSB taxes but also how to design, position, and communicate them as health-oriented fiscal policies.

## 1. Introduction

Sugar-sweetened beverages (SSBs) are a substantial source of added sugars and excess dietary energy and include soft drinks, sweetened juices, energy drinks, sweetened teas, ready-to-drink coffees, and other sugar-containing beverages. Regular consumption of SSBs has been linked to weight gain, obesity, type 2 diabetes, cardiovascular disease, and other diet-related non-communicable diseases (NCDs) [1,2,3,4,5]. Intake of SSBs has increased dramatically worldwide over the last few decades, especially in many low- and middle-income countries experiencing rapid urbanisation, income growth, dietary change, and expansion of commercial beverage markets [3,6,7]. This tendency is concerning as a high free-sugar intake is still inconsistent with public health recommendations, particularly the World Health Organisation (WHO) guideline on sugars intake for adults and children [8].

Strategies to reduce SSB consumption at the population level include front-of-pack warning labels, marketing restrictions to children, nutrition requirements in schools and other public venues, product reformulation, public education and access to safe drinking water [9,10]. As part of this overall policy package WHO strongly recommends the implementation of policies to tax SSBs to promote healthier diets [10]. Earlier WHO guidance suggests that a rise in retail price of at least 20% generated by taxation may result in considerable decreases in consumption [11,12]. These policies may contribute to the Sustainable Development Goal target 3.4, which aims, by 2030, to reduce by one third premature mortality from non-communicable diseases through prevention, treatment and promotion of mental health and well-being [13]. The World Bank and UNICEF both list SSB taxation as a policy tool that can benefit public health, generate revenue, and promote healthier food environments when adopted as part of a broader package of NCD-prevention strategies [14].

There is increasing evidence for the effectiveness of SSB taxes. Systematic reviews and meta-analyses of SSB taxes have shown that these levies are generally passed on to prices and are related with decreased purchases, sales or consumption of taxed beverages [15,16,17]. Research from Mexico revealed decreases in purchases of taxed beverages following the implementation of the national excise tax, and research from Berkeley, California, showed sales of taxed beverages declined by 9.6%, while water sales increased by 15.6% one year after tax implementation [18,19]. Evidence from Philadelphia indicated changes in beverage costs and sales after implementation of a sweetened beverage tax, taxed-beverage prices increased and sales volume declined by 51% [20]. Wider assessments of the political economy and behavioural consequences of SSB taxes also reveal that taxation can influence both consumer demand and industry actions, especially when taxes are visible, properly planned, and backed by public health communication [21,22].

The current evidence basis for the question of whether SSB taxes work is better developed than the evidence foundation for the question of how tax design shapes efficacy. SSB taxes vary widely across different geographies. Some are specific taxes, based on beverage volume, sugar content or quantity. Some are ad valorem taxes, based on a proportion of product value. Some are mixed designs, combining specific and ad valorem features [23]. These structures may differ in the strength of their effect on retail prices, their incentives for reformulation, the predictability of their effects on revenue, and the clarity of their transmission of tax-induced changes in price to consumers [21,24,25,26]. Emerging evidence from tiered soft drink taxes in Europe suggests the potential for tax design to affect industry reformulation and sales-weighted sugar content, although the majority of current information remains focused on a few high-income nations and specific policy settings [27].

Another crucial but less frequently examined design aspect is whether an SSB tax is expressly portrayed as a health-oriented policy. A tax declared to have a health objective may affect behaviour through more than just price adjustments [10,28]. Public debate, media coverage, legislative framing and health communication around such a tax may communicate that excessive SSB consumption is harmful and may enhance consumer knowledge of health hazards associated with sugar. Economic theory also shows that SSB taxes may correct for not only externalities (e.g., health-care expenses) but also internalities from incomplete nutrition knowledge, present bias, and self-control issues [28]. Thus, a health-oriented tax could act through both price and information channels, which may reinforce its relationship with lower SSB consumption.

The present analysis examines SSB tax implementation, tax design and soft-drink sales in different geographies between 2010 and 2024. Per capita soft-drink sales volume (litres per person per year) is used as market level proxy for SSB consumption. The study starts by estimating the average association between SSB taxation and per capita soft-drink sales volume using a multi-country quasi-experimental approach with staggered policy adoption. It next considers if this association varies by tax structure (specific, ad valorem and mixed) and whether taxes with a stated health objective are linked with greater reductions in per capita soft-drink sales volume (market level proxy for SSB consumption). We hypothesised that the implementation of a national SSB tax would be associated with lower per capita soft-drink sales volume (H1), that taxes with an explicit health objective would be associated with larger reductions than non-health-objective taxes (H2), and that the association would vary between ad valorem, mixed, and specific tax structures (H3). The conceptual framework guiding the analysis is presented in Supplementary Figure S1. This study adds to the evidence base on fiscal policy for health by examining both tax implementation and tax design and offers policy-relevant evidence for geographies aiming to design or enhance SSB taxation as part of NCD prevention measures.

## 2. Methods

### 2.1. Data

We developed a geography-year panel by merging Euromonitor soft-drink sales data, World Bank national SSB tax-policy data, and World Development Indicators macroeconomic data. The unit of analysis was geography-year. We acquired sales data for soft drinks from the Euromonitor International Passport database [29], categorised as “soft drinks,” for the period 2010–2024. The subcategories under soft-drink include: Asian speciality drinks, concentrates, energy drinks, juice, ready-to-drink (RTD) coffee, RTD tea, regular carbonates and sports drinks. Bottled water was excluded from our analysis. Euromonitor amalgamates many beverage categories into the “soft drinks” classification and does not give a consistent classification that permits the exclusion of 100% fruit juice or low-sugar variants across all geographies and years. Consequently, our SSB metric represents the wider soft drinks market as delineated by Euromonitor (excluding bottled water), potentially including certain 100% juices and low-sugar alternatives. Thus, our metric should be seen as representing the soft drinks (excluding bottled water), functioning as a consistent proxy for the demand for sugar-sweetened beverages in cross-geography studies. Euromonitor acquired these statistics through expert interviews and an analysis of several sources, including official publications, industry reports, scientific journals, and online sites.

National SSB tax policy information was taken from the World Bank SSB Tax Database, August 2023 version [23]. Tax-policy data were matched with Euromonitor geography-year sales data. The key policy variable was the first year of introduction of a national SSB tax. The information was used to identify tax design features including tax structure and if the tax had an explicit health objective. Tax structure was defined as specific, ad valorem, or mixed. The treatment variable captures national SSB taxes; subnational-only SSB taxes were not categorised as implementation of a national SSB tax.

The macroeconomic controls were collected from World Bank World Development Indicators [30]. The data included total population, gross domestic product (GDP) at purchasing power parity in current international dollar, and annual consumer price inflation. Population and GDP were entered in log form to minimise the effect of scale across geographies, and inflation was entered as the yearly consumer price inflation rate.

The primary difference-in-difference analysis excludes geographies that had already enacted an SSB tax at the start of the study period, as their pre-tax trends were not observable in the sample window. The raw data set included 96 geographies and 1536 geography-year observations for the years 2010 to 2025. The analysis was restricted to 2010–2024 excluding the 96 observations for 2025, due to lack of sale volume data for the year 2025 across all geographies. Next, we removed 19 geographies that had implemented an SSB tax by 2010 (285 observations), as there was no observable pre-tax period within the analytical window. Another 59 geography-year observations were dropped due to missing outcome or control data, removing three geographies altogether. The final estimation sample consists of 74 geographies and 1096 geography-year observations from 2010 to 2024. The geographies included in the final analytical sample and their treatment status are listed in Supplementary Table S6.

### 2.2. Measure

The dependent variable was per capita soft-drink sales volume, measured as litres per person per year. This was computed by dividing the total annual volume of soft-drink sales in litres by the total population in each geography in the relevant year. This measure was used as a market-level proxy for SSB consumption and did not directly measure individual beverage intake or added-sugar consumption.

The main treatment variable was if a national SSB tax was present in a geography-year. The first year of national SSB tax implementation was discovered for each geography using the World Bank SSB Tax Database. The treatment was marked as 1 for all years greater than or equal to the first tax-start year and 0 otherwise.

We examined the tax designs in two different ways. First the tax structure was divided into the following three groups: ad valorem, mixed and specific. Ad valorem taxes are based on value of the product, specific taxes are based on amount, volume or sugar related tax bases and mixed taxes combine ad valorem and specific tax elements. Second, the health objective was measured using the World Bank SSB Tax Database indication of whether the tax had an explicit health objective. Health-oriented SSB taxes were defined as taxes categorised with health objective = 1.

The control variables were population, GDP at purchasing power parity in current international dollars and yearly consumer price inflation. We added these parameters to account for geography-year changes in market size, economic conditions, and macroeconomic price situations that may affect beverage sales.

### 2.3. Analysis

**Baseline Regression:** SSB taxes were adopted at different points in time across geographies, generating a staggered policy-adoption setting. The primary analysis therefore used a staggered difference-in-difference (DID) model [31] to estimate the association between SSB taxation and per capita soft-drink sales volume. The primary estimator was Stata 18’s panel heterogeneous difference-in-difference regression-adjustment estimator, implemented with xthdidregress ra. This technique was chosen because traditional two-way fixed effect models might be sensitive to improper comparisons and weighting difficulties when treatment timing varies and treatment effects are heterogeneous [31,32]. Identification requires parallel trends in untreated potential outcomes and no policy anticipation or other differential shocks affecting treated geographies around the time of tax implementation [31,32].

The baseline model utilised never-treated geographies as the control group. The outcome model controlled for log population, log GDP at purchasing power parity and yearly consumer price inflation. Per capita soft-drink sales volume was the dependent variable. We clustered standard errors at the geography level. The treatment indicator was coded 1 if a national SSB tax was in place for a given geography-year, and 0 otherwise. The major metric of interest was the average treatment effect on the treated, representing the difference between observed post-tax per capita sales volume and the counterfactual level expected in the absence of the tax.

We produced event-study estimates using event time, which is the number of years relative to the initial year of implementation of the SSB tax, to investigate pre-policy trends and post-policy dynamics. Event time 0 is the year of implementation, negative numbers are the years before implementation and positive values are the years after implementation. We used five years prior to implementation and ten years after implementation to estimate dynamic treatment effects.

**Heterogeneity Analysis by Health Objective:** This analysis examines whether the explicit inclusion of a health objective in the SSB taxation improves its effectiveness. To do so, we used an auxiliary two-way fixed-effect interaction model, where the SSB tax indicator was interacted with a dummy variable indicating whether the policy explicitly incorporates a health objective.

In order to prevent geographies that did not have an SSB tax from entering the control group and thus confounding the estimated effect, the sample is restricted to geographies that have implemented an SSB tax. In this sample, geographies whose tax policies explicitly include a health objective are the treatment group, while those without a health objective are the control group.

**Heterogeneity Analysis by Tax Structure**: This analysis analyses if the impacts of SSB taxation vary across tax structures. Accordingly, separate regressions are estimated for each type of tax structure. For each subsample, geographies that have adopted the tax structure under discussion are allocated to the treatment group, whereas geographies that have never implemented the SSB tax are used as the control group.

For robustness, the main model was re-estimated with not-yet-treated geographies as the control group. As auxiliary evidence, we also estimated a conventional two-way fixed effect model with geography fixed effects, year fixed effects, macroeconomic controls, and geography-clustered standard errors.

Detailed model specifications and Stata code are provided in the Supplementary Materials in Stata replication code section.

## 3. Results

### 3.1. Descriptive Statistics

Descriptive statistics of the final estimation sample are shown in Table 1. Our sample comprised 74 geographies and 1096 geography-year observations from 2010 to 2024. Of these, 852 were untreated geography-year observations and 244 were post-tax observations. Examples of treated geographies included Thailand, Uganda, the United Arab Emirates, and the United Kingdom, while untreated controls included Singapore, Switzerland, Ukraine, and Uzbekistan (Supplementary Table S6). The average per capita soft-drink sales volume was 71.885 L per person in untreated observations and 62.527 L per person in post-tax data. The standard deviations were 38.694 and 47.203 respectively. This indicates that there is a lot of variance in per capita sales volume between geographies and years.

Also, macroeconomic controls were different across untreated and post-tax observations. The mean log population was 16.707 for untreated observations and 17.351 for post-tax observations. For untreated observations, the mean log GDP at purchasing power parity was 26.786 while for post-tax observations it was 27.129. Untreated observations had a mean annual consumer price inflation of 4.816% while post-tax observations had 5.391%. These disparities suggest that population, GDP and inflation should be included as controls in the outcome model.

### 3.2. Overall Association Between SSB Taxes and per Capita Sales Volume

Table 2 presents the estimated connection of SSB taxes with per capita soft-drink sales volume. In the baseline multi-period difference-in-difference model with never-treated geographies as the control group, SSB taxes were linked with a reduction of 7.286 L per person in per capita sales volume. This estimate was statistically significant at the 1 percent level.

The result remained robust when the control group was changed. Using not-yet-treated geographies as the control group, the estimated reduction was 7.157 L per person and remained statistically significant at the 1% level. The auxiliary conventional two-way fixed effect model also produced a negative estimate, with a reduction of 5.140 L per person, significant at the 5% level. Although the two-way fixed effect model was included only as supporting evidence, the direction of the estimate was consistent with the multi-period difference-in-difference results.

In the final sample, the average soft-drink sales volume per person was 69.801 L per capita. Thus, the baseline estimate of 7.286 L amounts to about 10.4% of the sample mean. This suggests that SSB taxes were related with an economically relevant drop in per capita soft-drink sales volume.

### 3.3. Dynamic Effects

**Figure 1:**
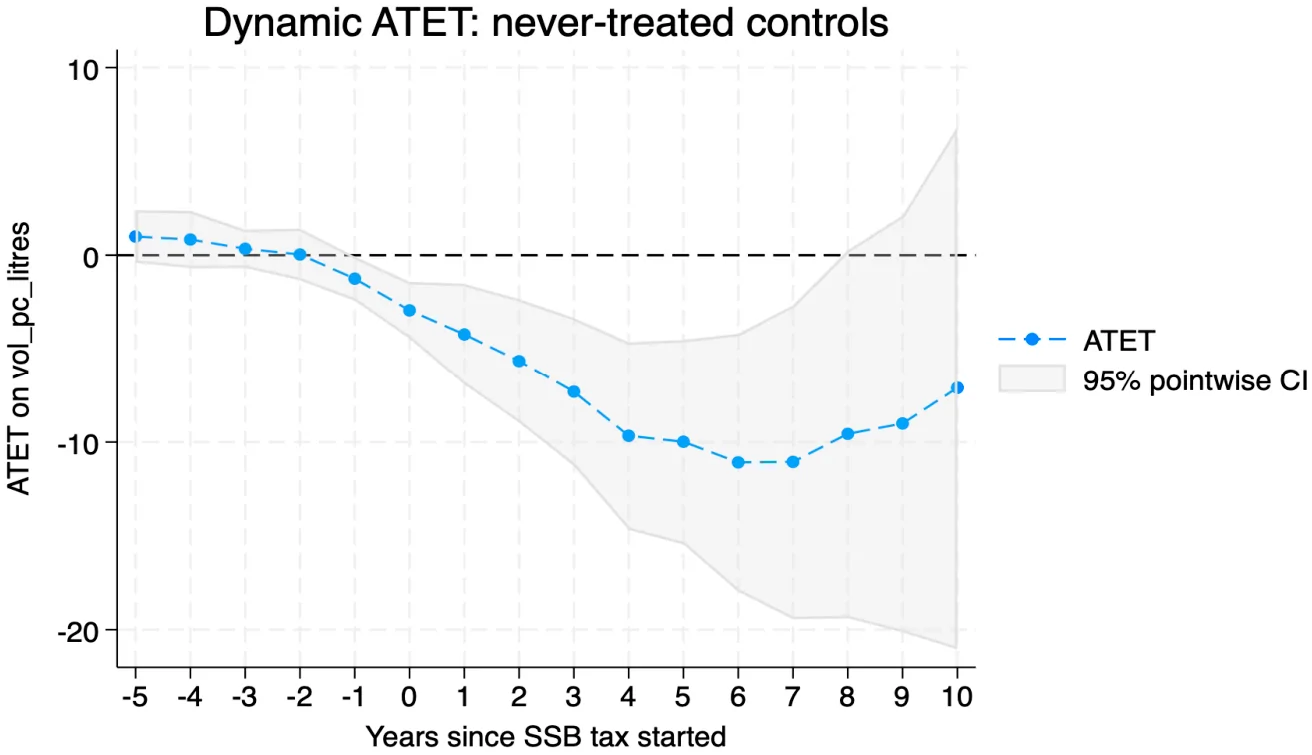
Dynamic ATET: never-treated controls.

The dynamic treatment estimates utilising the never-treated geographies as the control group are shown in Figure 1. Estimates indicate that the association of SSB taxation with per capita sales volume became more negative following tax introduction. The observed reduction in the year of implementation was 2.936 L per capita. The reduction increased to 4.221 L in the first year after implementation and 5.651 L in the second year after implementation.

The predicted reductions increased in the post-tax years. Between years four and seven post-implementation, the predicted reductions ranged from 9.664 to 11.056 L per person. Estimates for years 8 through 10 continued to be negative but with wider confidence intervals, reflecting more statistical uncertainty about longer-run impacts because fewer treated cohorts contributed to these later event-time estimates.

The pre-tax estimates five to two years before implementation were near to zero and statistically insignificant, indicating no persisting differential pre-tax trend across the extended pre-treatment timeframe. However, the estimate one year prior to implementation was negative and statistically significant, indicating that caution is required with respect to the timing and causal interpretation of the post-tax estimates. Therefore, the one-year pre-policy estimate should be used cautiously in terms of the timing of policy effects.

#### Heterogeneity by Health Objective

Table 3 presents two-way fixed effect interaction results to test if the association between SSB taxes and per capita soft-drink sales volume differs by explicit health objective. The analysis was restricted to 32 SSB tax-adopting geographies that had both pre-tax and post-tax observations, 23 with health-objective taxes and nine with non-health-objective taxes.

The estimated coefficient for non-health-objective taxes was −1.036 L per person and not statistically significant. The interaction term for health-objective taxes was −5.814 L per person and was statistically significant at the 5% level. This suggests that in tax-adopting geographies, there was an additional decrease in per capita soft-drink sales volume for taxes with an explicit health objective compared with those without a stated health objective.

The linear combination of the general tax effect and the health-objective interaction term showed that the total estimated effect of health-objective taxes was −6.850 L per person and was statistically significant at the 1% level. Taxes without a stated health objective may have been primarily introduced to raise government revenue, to broaden the excise-tax base, or to achieve other fiscal objectives.

### 3.4. Heterogeneity by Tax Structure

Table 4 presents multi-period difference-in-difference estimates per tax structure. Ad valorem taxes were related with a drop of 9.890 L per person in per capita soft-drink sales volume and were statistically significant at the 1% level. Mixed taxes were statistically significant at the 5% level and related with a reduction of 10.747 L per person. The specific taxes were related with a reduction of 3.437 L per person (statistically significant at 10% level, 95% confidence interval including 0).

The point estimates were negative for all three tax structures, implying that ad valorem, mixed, and specific taxes were each linked with lower per capita soft-drink sales volume at varying levels of statistical significance. However, the formal equality tests of the auxiliary two-way fixed effect model did not find statistically significant differences across tax structures. The *p* value for difference between ad valorem and mixed taxes was 0.883, the *p* value for difference between ad valorem and specific taxes was 0.440 and the *p* value for difference between mixed and specific taxes was 0.672.

These results suggest that SSB taxes were related with decreases in per capita sales volume under different tax designs, but the data do not support a conclusion that one tax structure was statistically superior to another in this sample.

## 4. Discussion

This study presents cross-geography evidence that SSB taxation is associated with lower per capita soft-drink sales volume, used in this study as a market-level proxy for SSB consumption. Using a multi-period difference-in-difference approach for 74 geographies from 2010 to 2024, we found that SSB taxes were associated with an average decrease of 7.286 L per person in soft-drink sales volume, which is approximately 10.4% of the sample mean. The result is robust when an alternative control group of not-yet-treated geographies is utilised and the auxiliary two-way fixed effect model shows the same negative direction. Results suggest that SSB taxes may contribute to lower per capita soft-drink sales volume (used in this study as a market-level proxy for SSB consumption). However, the outcome may include some 100% juices and low sugar products, so this limitation should be considered before the findings are interpreted as indicating reduced added sugar intake or lower NCD risk.

Our results on the magnitude and the direction of effect are in line with the broader research on SSB taxes. Evidence from systematic reviews and meta-analyses indicates that implemented SSB taxes are largely passed on to consumers in prices and are associated with lower sales of taxed beverages [15,16]. Individual policy settings also support this pattern. For example, work in Mexico and Berkeley showed reductions in purchases or sales of taxed beverages after introduction of the SSB tax [18,19]. This study contributes to this literature by examining SSB tax implementation across a wider collection of geographies and by focusing not only on whether SSB taxes are linked with reduced per capita soft-drink sales volume (used in this study as a market-level proxy for SSB consumption), but also on whether the strength of the connection is sensitive to tax design factors.

The dynamic results imply that the association between SSB taxes and per capita soft-drink sales volume strengthened over time. Estimated reductions were observed in the implementation year and increased over subsequent years, particularly between the fourth and seventh post-implementation years. Likely, the effect of SSB taxes may not be immediate or operate only through increases in pricing. It may take time for firms to modify prices, packaging, marketing or product portfolios and it may take time for consumers to adjust their purchase behaviour. At the same time, the negative and significant estimate one year prior to implementation should be read with caution. It also may indicate anticipation of policy, since SSB taxes often are publicly discussed, published, or communicated in advance of the actual tax-start date and that may influence corporate and consumer behaviour [26]. This is consistent with quasi-experimental research showing that policy announcements and elections may affect market responses before tax implementation [26,33].

One notable contribution from this analysis is the finding that taxes with an explicit health objective were linked with larger reductions in per capita soft-drink sales volume than taxes without such an objective. For the health-objective model, non-health-objective taxes were not substantially associated with lower per capita soft-drink sales volume, but health-objective taxes had a significant overall reduction. This implies that the effectiveness of SSB taxation may not just depend on the existence of a tax, but also on the way the policy is structured, communicated and perceived by the public. A tax framed primarily as a revenue measure may have a weaker behavioural signal than one clearly presented as a public-health strategy; policy communication and political processes may influence consumer behaviour even before implementation [10,28,33]. Framing the tax in a health context could improve awareness of the hazards of consuming sugar, lend legitimacy to the tax and strengthen the behavioural effect of price hikes. This explanation is also compatible with economic arguments that SSB taxes may address not only health-care externalities but also internalities related to imperfect dietary information, present bias, and self-control issues [28].

The findings by tax structure also offer useful policy insight. All ad valorem, mixed and specific taxes were linked with reductions in per capita soft-drink sales volume at varied degrees of statistical significance. Point estimates were bigger for ad valorem and mixed taxes than for specific taxes, but formal equality tests did not find statistically significant differences among tax types. Therefore, these results should not be construed as evidence of one tax structure being superior in general. Instead, they argue that SSB taxes can be helpful in many configurations, but careful design is still required. The design of the tax system can determine the magnitude of the effect of taxes on prices paid by consumers, the predictability of revenues, whether enterprises have incentives to reformulate products, and if the tax burden shifts over time with inflation. Evidence from tiered soft-drink levies in Europe also illustrates the importance of design factors in shaping industry reactions and sales-weighted sugar content [27].

These findings have direct implications for fiscal policy for health. First, they endorse WHO-recommended initiatives to implement or improve SSB taxes as part of NCD preventive programmes [10]. Second, they argue that governments should not view SSB taxes merely as a revenue instrument. Explicitly framing them as health policies may enhance their public-health impact. Third, adoption should be paired with monitoring of tax pass-through, beverage prices, sales volume, product reformulation, and replacement to untaxed beverages [34]. Finally, the taxing of SSB needs to be linked into broader nutrition and NCD prevention packages, including front-of-pack labelling, limits on marketing to children, school food policy and public health communication. Also, SSB taxation should be considered as part of wider NCD-prevention initiatives that encourage physical activity and minimise sedentary behaviour [35]. Although fiscal policy alone is unlikely to totally reverse rising SSB demand, it can be an important component of a comprehensive approach to reduce added-sugar consumption and enhance public health.

There are equality and implementation concerns with SSB taxes as well. Although their financial burden may be regressive relative to income, lower-income groups may also experience greater health benefits [36]. Lower soft-drink sales may not translate proportionally into lower total sugar intake as consumers may substitute towards untaxed sugary products, or purchase beverages outside the taxed jurisdiction [17,34]. Price-based strategies may also not address social or emotional coping-associated consumption. Equity and public trust may be improved by reinvesting tax revenue transparently into safe drinking water, access to healthy food, health services, and disadvantaged communities [37].

Strengths of the study include its broad cross-geography and longitudinal coverage, use of a modern staggered-adoption DID estimator, linkage of independent international data sources, robustness checks, provision of detailed analytical code and its focus not only on SSB tax implementation but also on tax-design features, including stated health objective and tax structure.

Several limitations should be acknowledged. First of all, the result was based on the categories of soft-drink market from Euromonitor, excluding bottled water. These categories are a consistent metric across geographies but may contain certain 100% fruit juice or low-sugar version that are not necessarily directly targeted by SSB taxes. This is particularly true in the European Union countries, where fruit juices by law cannot contain added sugar, unlike some juice drinks or nectars [38]. These products may not have been subject to SSB taxation or may have responded differently than taxed beverages, and their inclusion may have diluted the observed association, may have led to outcome measurement error, and may have dampened the projected tax impacts. However, substitution between beverage categories means that the direction of any resulting bias cannot be determined with certainty. Second, per capita sales volume is a market-level proxy of SSB consumption and does not reflect individual dietary intake. Third, the analysis concentrated on national implementation of SSB taxes; subnational-only taxes were not coded as national taxes, which may understate policy exposure in places where significant local SSB taxes are in place. Fourth, the tax-policy data covered tax timing and chosen design aspects, but the analysis did not directly assess tax rates, tax pass-through, enforcement, earmarking, indexation, or product-level reformulation. Fifth, health-objective analysis used an auxiliary TWFE interaction model and hence may be more sensitive to heterogeneous treatment effects than the primary multi-period DID estimates. Other limitations are the significant pre-treatment estimate of one-year, heterogeneity in tax systems and socioeconomic contexts across different geographies, the possibility of residual confounding due to co-occurring policies and market shocks, and limited statistical power in smaller heterogeneity groups, in particular for mixed taxes.

Nonetheless, this study contributes to the evidence base by providing quasi-experimental evidence on the design and implementation of SSB taxes across geographies. Our findings imply that SSB taxes are associated with large reductions in SSB consumption, and that health-oriented policy framing may strengthen this association. Overall, the findings suggest that governments need to examine not only whether to impose SSB taxes, but how to design, communicate and integrate them in larger fiscal policy for health and NCD prevention efforts.

## 5. Conclusions

Using a cross-geography quasi-experimental design, we examine the association between SSB taxation, tax design and per capita soft-drink sales volume as a market-level proxy for SSB consumption across 74 geographies from 2010 to 2024. Results suggest that SSB taxes were associated with a significant reduction in per capita soft-drink sales volume, equivalent to roughly 10.4% of the sample mean. Taxes explicitly designed with public-health purpose were related with higher reductions than those introduced without a clear health focus, suggesting that the policy design and communication may enhance the public-health impact of fiscal measures. Ad valorem, mixed and specific taxes were all related with reductions in per capita soft-drink sales volume. However, no single tax structure demonstrated a statistically significantly advantage over the others within our sample. Overall, these findings provide robust empirical support for well-designed, clearly health-focused SSB taxation as an effective instrument within broader fiscal policy for health and NCD prevention efforts, in line with WHO recommendations and the target of Sustainable Development Goal 3.4. However, cautious interpretation of the results is required because the outcome is market-level proxy for SSB consumption, and possible residual confounding and cross-geography heterogeneity.

## Figures and Tables

**Table 1:** Descriptive statistics.

Variable	Sample	Obs.	Mean	Std. Dev.	Min.	Max.
Soft drink consumption per person	Untreated	852	71.89	38.69	2.98	205.81
Soft drink consumption per person	Post-tax	244	62.53	47.20	5.20	209.79
Log population	Untreated	852	16.71	1.46	14.09	21.07
Log population	Post-tax	244	17.35	1.25	14.73	21.10
Log GDP PPP	Untreated	852	26.79	1.45	23.87	31.27
Log GDP PPP	Post-tax	244	27.13	1.23	24.72	30.42
Inflation rate	Untreated	852	4.82	12.20	−3.75	221.34
Inflation rate	Post-tax	244	5.39	6.69	−2.54	49.72

The post-tax period includes all years after an SSB tax was introduced, including the year the tax started.

**Table 2:** Effect of SSB taxes on per capita soft-drink consumption.

Variable	Baseline: Never-Treated Controls	Robustness: Not-Yet-Treated Controls	Conventional TWFE
SSB tax (tax_treat)	−7.286 ***	−7.157 ***	−5.140 **
	(2.21)	(2.17)	(2.45)
Controls	Yes	Yes	Yes
Year fixed effects	Handled by method	Handled by method	Yes
Geography fixed effects	Handled by method	Handled by method	Yes
geography-clustered robust SE	Yes	Yes	Yes
Observations	1096	1096	1096
Geographies	74	74	74

Standard errors, clustered at the geography level, are shown in parentheses. *** and ** indicate statistical significance at the 1% and 5% levels, respectively. The baseline and robustness models were estimated using xthdidregress ra; the TWFE model is included only as a supporting analysis.

**Table 3:** Health-objective heterogeneity: TWFE interaction model.

Item	Statistical Expression	Coefficient	Std. Error	*p*-Value	Interpretation
Effect of non-health-objective taxes	beta_1	−1.036	1.628	0.529	Average effect of non-health-objective SSB taxes; not significant
Additional effect of health objective	beta_2	−5.814 **	2.671	0.037	Additional decline relative to non-health-objective taxes
Total effect of health-objective taxes	beta_1 + beta_2	−6.850 ***	2.218	0.004	Overall effect after health-objective tax implementation

The model accounts for geography and year differences and adjusts for population, GDP at purchasing power parity, and inflation. Standard errors are clustered at the geography level. The test for the additional health-objective effect was statistically significant (F = 4.74, *p* = 0.0372). *** and ** indicate significance at the 1% and 5% levels, respectively.

**Table 4:** Tax-structure heterogeneity: multi-period DID results.

Tax Structure	Treated Geographies	Obs.	Clusters	ATET	Std. Error	*p*-Value	95% CI
Ad valorem	12	799	54	−9.890 ***	3.733	0.008	[−17.206, −2.574]
Mixed	5	695	47	−10.747 **	4.614	0.02	[−19.790, −1.704]
Specific	14	830	56	−3.437 *	2.024	0.089	[−7.404, 0.529]

Separate multiperiod difference-in-difference models were used to evaluate each tax structure. For each model, we compared geographies with that tax structure to geographies that did not implement an SSB tax across the study period. The models are controlled for population, GDP at purchasing power parity and inflation. *, **, and *** denote significance at the 10%, 5%, and 1% levels, respectively.

## Data Availability

The data in this study for sales volume of sugar-sweetened beverages (SSBs) were obtained from Euromonitor International Passport which are not publicly available due to third-party restrictions. So, the full replication would require a licenced access of Euromonitor Passport. Data on SSB tax were gathered from the World Bank Global SSB Tax Database. Macroeconomic indicators (population, GDP in purchasing power parity, and inflation) were sourced from the World Bank World Development Indicators. These World Bank data sources are publicly available via the respective World Bank data portals. The analytic code used for data cleaning and analysis in this study is provided in the Supplementary File. Non-proprietary data components are available from the corresponding author upon reasonable request.

## Supplementary Materials

The following supporting information can be downloaded at https://www.mdpi.com/article/10.3390/ijerph23080985/s1, Figure S1: Conceptual framework for the study; Figure S2: Dynamic atet; Table S1: Ssb tax implementation year, tax structure, and health objective; Table S2: Beverage categories included/excluded, table Euromonitor international passport; Table S3: Data sources and variable construction; Table S4: Analytical sample construction summary; Table S5: Descriptive statistics; Table S6: Final analytical sample of 74 geographies by Ssb tax implementation year, treatment status, and tax structure; Table S7: Effect of SSB taxes on per capita soft-drink consumption; Table S8: Health-objective heterogeneity: TWFE interaction model; Table S9: Tax-structure heterogeneity: multi-period DID Results.
