# Supplementary material for: Fiscal Policy for Health: Sugar-Sweetened Beverage Tax Design and Soft Drink Consumption Across Geographies, 2010–2024
Source: Int J Environ Res Public Health. 2026 Jul 29;23(8):985. doi: 10.3390/ijerph23080985 (PMC13512422; doi:10.3390/ijerph23080985)
Supplement: Supplementary file 1 [file ijerph-23-00985-s001.zip › ijerph-4425051-supplementary.pdf]

Supplementary Materials

Title: Fiscal Policy for Health: Sugar-Sweetened Beverage Tax Design and Soft Drink Consumption Across Geographies, 2010-2024

Contents

1. Overview of the study, and SSB taxes in the World Bank Global SSB Tax Database 3

1.1 Conceptual framework for the study 3

2. Beverage categories and Euromonitor Passport definitions 9

3. Data sources, sample construction, and variable construction 11

4. Stata replication code 13

5. Empirical Results: 32

5.1 Data Sources and Sample Construction 32

5.2 Empirical Specification 39

5.3 Baseline Regression Results 40

5.4 Dynamic Effects and Policy Anticipation 41

5.5 Heterogeneity by Health Objective 42

5.6 Heterogeneity by Tax Structure 43

References 45

1. Overview of the study, and SSB taxes in the World Bank Global SSB Tax Database

1.1 Conceptual framework for the study

FIGURE S1 CONCEPTUAL FRAMEWORK FOR THE STUDY

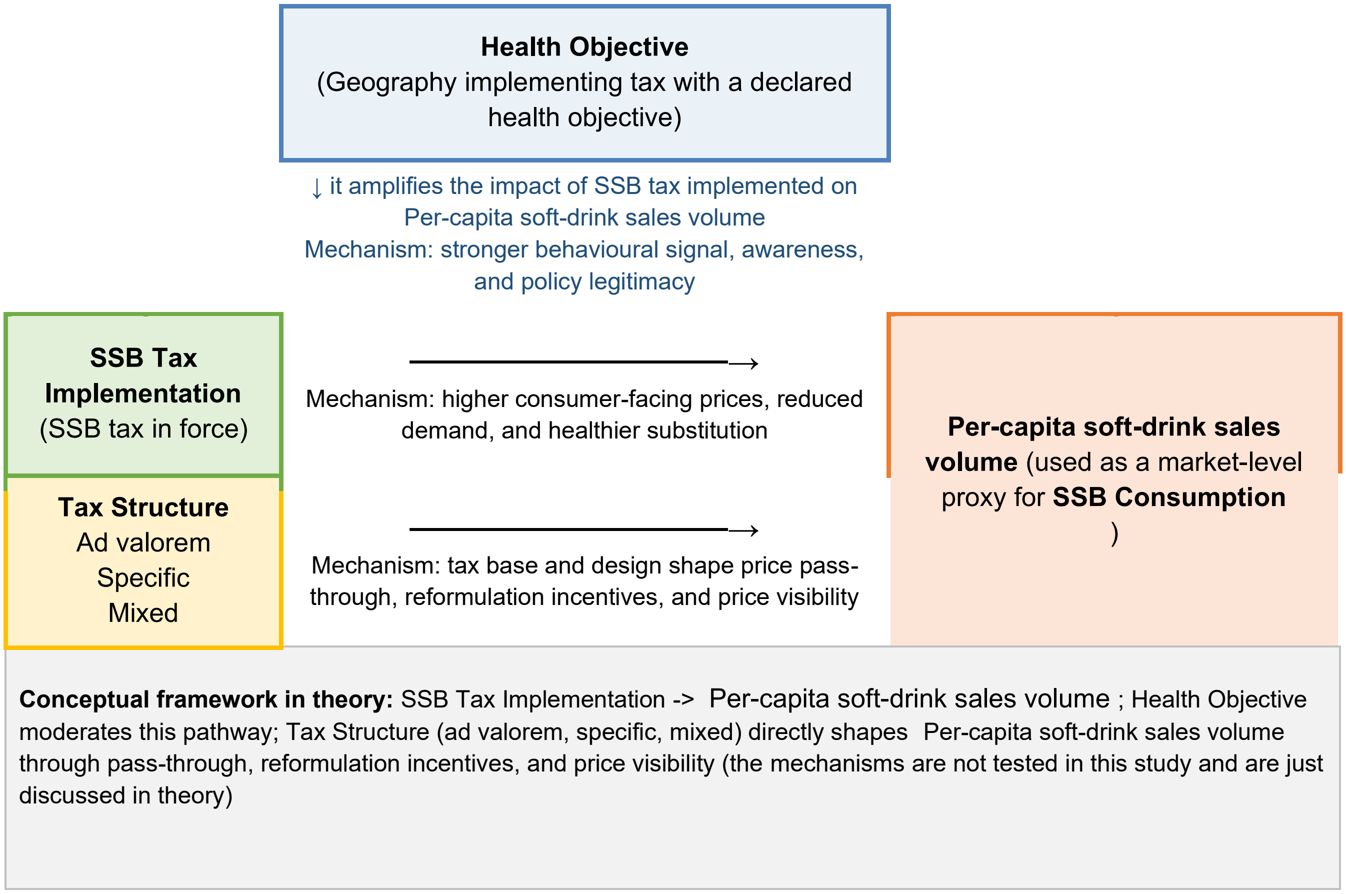

Table S1 provides an overview of national SSB tax policies recorded in the World Bank Global SSB Tax Database, August 2023 version.

TABLE S1 SSB TAX IMPLEMENTATION YEAR, TAX STRUCTURE, AND HEALTH OBJECTIVE

Geography	Tax implementation year	Tax structure	health objective
American Samoa	2001	Specific	No
Argentina	1996	Ad valorem	No
Azerbaijan	2019	Specific	No
Bahrain	2017	Ad valorem	Yes
Bangladesh	2012	Ad valorem	No
Barbados	2015	Ad valorem	Yes
Belgium	2009	Specific	Yes
Belize	2000	Specific	No
Benin	2011	Ad valorem	No
Bermuda	2018	Ad valorem	Yes
Bolivia	2016	Specific	No
Brazil	1965	Ad valorem	No
Brunei Darussalam	2017	Specific	Yes
Burkina Faso	1995	Ad valorem	Yes
Burundi	2012	Mixed	No
Cabo Verde	2019	Ad valorem	No
Cambodia	2003	Ad valorem	Yes
Cameroon	2019	Mixed	No
Central African Republic	2019	Ad valorem	No
Chad	2019	Ad valorem	No
Chile	2014	Ad valorem	Yes

Congo, DR	2018	Ad valorem	No
Cook Islands	2014	Specific	Yes
Costa Rica	2001	Specific	No
Croatia	1994	Specific	Yes
Côte d'Ivoire	2018	Ad valorem	No
Dominica	2015	Mixed	Yes
Ecuador	2016	Mixed	Yes
Egypt	2016	Ad valorem	No
El Salvador	2010	Mixed	Yes
Equatorial Guinea	2020	Specific	No
Eritrea	2001	Mixed	No
Ethiopia	2003	Ad valorem	Yes
Fiji	1986	Specific	Yes
Finland	2001	Specific	Yes
France (and overseas departments and regions)	2012	Specific	Yes
French Polynesia	2004	Specific	Yes
Gabon	2013	Ad valorem	No
Gambia, The	2010	Specific	No
Ghana	2014	Ad valorem	No
Grenada	2023	Ad valorem	Yes
Guatemala	2002	Specific	Yes
Guinea-Bissau	2022	Mixed	No
Honduras	2020	Specific	Yes
Hungary	2011	Specific	Yes
India	2017	Ad valorem	Yes
Ireland	2018	Specific	Yes
Isle of Man	2019	Specific	Yes
Kenya	2015	Specific	No

Kiribati	2014	Ad valorem	Yes
Lao PDR	2005	Ad valorem	No
Latvia	2000	Specific	Yes
Liberia	2011	Specific	No
Madagascar	2016	Mixed	No
Malaysia	2019	Specific	Yes
Maldives	2017	Mixed	Yes
Mali	2005	Ad valorem	No
Marshall Islands	1989	Mixed	Yes
Mauritania	2014	Ad valorem	No
Mauritius	2013	Specific	Yes
Mexico	2014	Mixed	Yes
Monaco	2012	Specific	Yes
Montenegro	2001	Specific	No
Morocco	2019	Specific	Yes
Mozambique	2017	Specific	No
Nauru	2007	Ad valorem	Yes
Nepal	2002	Specific	No
Netherlands, The Kingdom of the	1992	Specific	No
New Caledonia	2017	Ad valorem	Yes
Nicaragua	2019	Ad valorem	No
Niger	2015	Ad valorem	No
Nigeria	2021	Specific	Yes
Niue	1969	Ad valorem	Yes
Northern Mariana Islands	1995	Specific	No
Oman	2019	Ad valorem	Yes
Pakistan	2005	Ad valorem	No

Palau	2003	Mixed	No
Panama	1995	Ad valorem	No
Paraguay	1992	Ad valorem	No
Peru	1999	Ad valorem	Yes
Philippines	2018	Specific	Yes
Poland	2021	Specific	Yes
Portugal	2017	Specific	Yes
Qatar	2019	Ad valorem	Yes
Romania	2023	Ad valorem	Yes
Russian Federation	2023	Specific	Yes
Rwanda	2019	Ad valorem	No
Saint Helena	2014	Specific	Yes
Saint Kitts and Nevis	2010	Ad valorem	No
Saint Vincent and the Grenadines	2007	Ad valorem	No
Samoa	1998	Specific	Yes
Sao Tome and Principe	1976	Ad valorem	No
Saudi Arabia	2017	Ad valorem	Yes
Senegal	2001	Ad valorem	No
Seychelles	2019	Specific	Yes
South Africa	2018	Specific	Yes
South Sudan	2009	Ad valorem	No
Spain	2021	Ad valorem	Yes
Sri Lanka	2018	Specific	Yes
Suriname	2006	Specific	No
Tajikistan	2018	Specific	No
Tanzania, United Republic of	2018	Specific	No

Thailand	2017	Mixed	Yes
Timor-Leste, Dem. Republic of	2023	Specific	Yes
Togo	2019	Ad valorem	No
Tonga	2013	Specific	Yes
Tunisia	2018	Ad valorem	No
Tuvalu	2009	Ad valorem	Yes
Türkiye	2002	Ad valorem	No
Uganda	2014	Mixed	No
United Arab Emirates	2017	Ad valorem	Yes
United Kingdom	2018	Specific	Yes
Uruguay	1990	Specific	No
Vanuatu	2002	Ad valorem	Yes
Vanuatu	2015	Specific	Yes
Wallis and Futuna	2017	Ad valorem	No
Zambia	2018	Specific	No
Zimbabwe	2022	Mixed	Yes
USA	No national tax	Subnational-only taxes not included	Not coded nationally

2. Beverage categories and Euromonitor Passport definitions

Soft-drink sales volume was operationalised by Euromonitor Passport soft-drinks sales volume excluding bottled water. The categories considered were totalled to derive the total annual sales volume for each geography-year. Category classifications were adapted from Euromonitor Passport category definitions and harmonised with the categories used in the present analysis.

TABLE S2 BEVERAGE CATEGORIES INCLUDED/EXCLUDED, TABLE EUROMONITOR INTERNATIONAL PASSPORT

**Passport category**	**Included?**	**Category definition**
Soft Drinks	No	Parent aggregation of carbonates, fruit/vegetable juice, bottled water, functional drinks, concentrates, RTD tea, RTD coffee, and Asian speciality drinks. The parent category was not directly used because study categories were selected and summed.
Bottled Water	No	Packaged drinking water, including still, carbonated, flavoured, functional, and bulk bottled water. Bottled water was excluded from the SSB-related sales-volume measure.
Asian Speciality Drinks	Yes	Traditional Asian drinks not included in RTD tea or juice drinks, such as bird’s nest, tamarind juice, ginger, lemongrass, roselle, jelly drinks, sugar cane drinks, vinegar drinks, and lactic acid drinks.
Concentrates	Yes	Aggregation of liquid concentrates and powder concentrates that are diluted with water before consumption, including syrups, squashes, dilutables, powdered drinks, and similar products.
Energy Drinks	Yes	Functional drinks designed to boost energy levels, often lightly carbonated and commonly containing caffeine, B vitamins, taurine, glucuronolactone, guarana, or ginseng. Glucose-based energy beverages are included.
Juice	Yes	Still packaged juice obtained from fruits or vegetables by mechanical processes, reconstituted or fresh. The category may include 100% juice, nectars, juice drinks, and coconut or other plant waters.
Ready-to-Drink (RTD) Coffee	Yes	Packaged ready-to-drink coffee consumed hot or cold and made using brewed coffee or coffee extract. Coffee-flavoured milk drinks primarily targeting children are excluded by Euromonitor.
Ready-to-Drink (RTD) Tea	Yes	Packaged products based on brewed tea or tea extract. Products may be sweetened or unsweetened, carbonated or still, and may contain juice.

Regular Carbonates	Yes	Regular sweetened, non-alcoholic carbonated drinks. Carbonated products containing fruit juice are included unless tea-based or energy drinks; carbonated bottled water is excluded.
Sports Drinks	Yes	Isotonic, hypotonic, and hypertonic drinks designed to provide fluids, electrolytes, carbohydrates, or a combination of these during or after exercise and heat exposure.

3. Data sources, sample construction, and variable construction

TABLE S3 DATA SOURCES AND VARIABLE CONSTRUCTION

Variable	Source	Construction of variables
Per-capita soft-drink sales volume	Euromonitor International Passport; World Bank population	Total annual soft-drink sales volume excluding bottled water divided by total population in each geography-year; measured in litres per person per year.
SSB tax implementation year	World Bank Global SSB Tax Database, August 2023	First year in which a national SSB tax was implemented in each geography.
Treatment indicator	Constructed by authors	Coded 1 for geography-years in which Year >= first national SSB tax implementation year; coded 0 otherwise.
Tax structure	World Bank Global SSB Tax Database, August 2023	Classified as specific, ad valorem, or mixed.
Explicit health objective	World Bank Global SSB Tax Database, August 2023	Coded 1 where the database identified an explicit health objective; otherwise coded 0.
Population	World Bank World Development Indicators	Total population; used to compute per-capita sales volume and entered in logarithmic form in regression models.
GDP at PPP	World Bank World Development Indicators	GDP at purchasing power parity in current international dollars; entered in logarithmic form in regression models.
Inflation	World Bank World Development Indicators	Annual consumer price inflation rate; included as a macroeconomic control.

TABLE S4 ANALYTICAL SAMPLE CONSTRUCTION SUMMARY

Step	Sample size	Description
Original data file	96 geographies; 1,536 geography-year observations	Balanced panel before DID exclusions.
Main DID-eligible geographies	Geographies never taxed nationally or first nationally taxed after 2010	Geographies taxed before or in 2010 were excluded because pre-tax trends were not observable within the sample window.

Final complete-case estimation sample	74 geographies; 1,096 geography-year observations	Complete observations for outcome, treatment indicator, log population, log GDP PPP, inflation, geography ID, and year.
Untreated observations	852 geography-year observations	Geography-years without a national SSB tax in force.
Post-tax observations	244 geography-year observations	Geography-years with a national SSB tax in force.

Note: The 2010–2025 period refers to the original input dataset, whereas the final analytical sample covers 2010–2024.

4. Stata replication code

```
version 18.0
clear all
set more off
set linesize 255

*******************************************************************************
* SSB TAXES AND PER CAPITA SOFT DRINK CONSUMPTION
* Reproduction code for the final empirical section
*
* Software: Stata 18 or later
* Input:    data.dta
* Output:   output/ (logs, tables, figures, and saved estimates)
*
* Reproduction instructions:
*   1. Place this do-file and data.dta in the same folder.
*   2. In Stata, change the working directory to that folder.
*   3. Run: do "SSB_Tax_Empirical_Submission.do"
*******************************************************************************

* The project root is the current Stata working directory. This keeps the code
* portable and avoids user-specific absolute paths.
global PROJECT "` c(pwd)'"

capture confirm file "${PROJECT}/data.dta"
if _rc {
    display as error "data.dta was not found in ${PROJECT}."
    display as error "Change Stata's working directory to the project folder and rerun."
```

```
    exit 601
}

capture mkdir "${PROJECT}/output"
capture log close _all
log using "${PROJECT}/output/SSB_Tax_Empirical_Submission.log", replace text

******************************************************************************
* 1. DATA VALIDATION AND BASELINE SAMPLE
******************************************************************************

use "${PROJECT}/data.dta", clear

describe Geography Category Year tax_start_year vol_pc_litres ///
    Population_total GDP_PPP_current_intl_usd ///
    Inflation_consumer_prices_annual health_obj_active tax_structure

isid Geography Year, sort
assert inrange(Year, 2010, 2025)
assert Category == "Total Soft Drinks" if !missing(Category)

egen long cid = group(Geography), label
label variable cid "Country identifier"

* The first tax year is defined at the country level. Missing values identify
* countries that never adopted an SSB tax during the observed policy history.
bysort cid: egen int first_tax_year = min(tax_start_year)
label variable first_tax_year "First SSB tax start year"
```

```
gen byte ever_taxed = !missing(first_tax_year)
label variable ever_taxed "Country ever adopted an SSB tax"

gen byte already_taxed_at_baseline = ever_taxed & first_tax_year <= 2010
label variable already_taxed_at_baseline "Tax started in or before 2010"

display as text "Countries excluded because the tax started in or before 2010:"
preserve
  keep if already_taxed_at_baseline
  bysort cid: keep if _n == 1
  list Geography first_tax_year, noobs abbreviate(24)
restore

* Countries already treated at the start of the panel have no observable
* pre-treatment period and are excluded from the DID estimation sample.
keep if missing(first_tax_year) | first_tax_year > 2010

gen byte tax_treat = ever_taxed & Year >= first_tax_year
label variable tax_treat "SSB tax in force"

* Population and GDP enter in logarithms; inflation remains in annual percent.
gen double ln_pop = ln(Population_total) if Population_total > 0
label variable ln_pop "Log population"

gen double ln_gdp_ppp = ln(GDP_PPP_current_intl_usd) ///
  if GDP_PPP_current_intl_usd > 0
label variable ln_gdp_ppp "Log GDP PPP"

gen double inflation = Inflation_consumer_prices_annual
```

```
label variable inflation "Annual consumer price inflation"

* Complete-case sample used by all baseline specifications.
keep if !missing(vol_pc_litres, tax_treat, ln_pop, ln_gdp_ppp, ///
    inflation, cid, Year)

xtset cid Year

* Reproduction checks for the final dataset supplied with the paper.
assert _N == 1096
egen byte country_tag = tag(cid)
quietly count if country_tag
assert r(N) == 74
drop country_tag

quietly count if tax_treat == 0
assert r(N) == 852
quietly count if tax_treat == 1
assert r(N) == 244

summarize vol_pc_litres ln_pop ln_gdp_ppp inflation
tabulate first_tax_year, missing
tabulate tax_treat, missing

tempfile analysis_sample
save `analysis_sample', replace

*******************************************************************************
* 2. TABLE 1: DESCRIPTIVE STATISTICS
```

```
*******************************************************************************

tempfile descriptive_results
tempname descpost
postfile ` descpost' str45 variable str12 sample double N mean sd min max ///
    using ` descriptive_results', replace

foreach v in vol_pc_litres ln_pop ln_gdp_ppp inflation {
    local vlabel "` v'"
    if "` v'" == "vol_pc_litres" local vlabel "Per capita soft drink consumption"
    if "` v'" == "ln_pop"        local vlabel "Log population"
    if "` v'" == "ln_gdp_ppp"    local vlabel "Log GDP PPP"
    if "` v'" == "inflation"     local vlabel "Inflation rate"

    forvalues d = 0/1 {
        local slabel "Untreated"
        if ` d' == 1 local slabel "Post-tax"

        quietly summarize ` v' if tax_treat == ` d'
        post ` descpost' ("` vlabel'") ("` slabel'") ///
            (r(N)) (r(mean)) (r(sd)) (r(min)) (r(max))
    }
}
postclose ` descpost'

preserve
    use ` descriptive_results', clear
    format mean sd min max %12.3f
    export delimited using "${PROJECT}/output/Table1_descriptive_statistics.csv", replace
```

```
    list, noobs abbreviate(24)
restore

******************************************************************************
* 3. TABLE 2: BASELINE AND ROBUSTNESS ESTIMATES
******************************************************************************

tempfile baseline_results ptrends_results dynamic_results
tempname basepost ptpost dynpost

postfile ` basepost' str34 model double estimate se statistic p ll ul N clusters ///
    using ` baseline_results', replace
postfile ` ptpost' str34 model double chi2 df p ///
    using ` ptrends_results', replace
postfile ` dynpost' int exposure double atet se z p ll ul ///
    using ` dynamic_results', replace

* 3.1 Main multi-period DID: regression adjustment and never-treated controls.
use ` analysis_sample', clear
xtset cid Year

xthdidregress ra ///
    (vol_pc_litres ln_pop ln_gdp_ppp inflation) ///
    (tax_treat), ///
    group(cid) controlgroup(never) ///
    cohortvar(cohort_year_never, replace) nolog
estimates store did_ra_never
estimates save "${PROJECT}/output/did_ra_never.ster", replace
```

```
* Joint test of all cohort-specific pre-treatment ATETs.
estat ptrends
post `ptpost' ("Multi-period DID: never treated") ///
    (r(chi2)) (r(df)) (r(p))

estat aggregation, overall
matrix M_never = r(table)
post `basepost' ("Baseline: never-treated controls") ///
    (M_never[1,1]) (M_never[2,1]) (M_never[3,1]) ///
    (M_never[4,1]) (M_never[5,1]) (M_never[6,1]) ///
    (e(N)) (e(N_clust))

* Event-time effects reported in Figure 1 and the accompanying discussion.
estat aggregation, dynamic(-5/10)
matrix M_dynamic = r(table)
forvalues j = 1/16 {
    local event_time = `j' - 6
    post `dynpost' (`event_time') ///
        (M_dynamic[1,`j']) (M_dynamic[2,`j']) (M_dynamic[3,`j']) ///
        (M_dynamic[4,`j']) (M_dynamic[5,`j']) (M_dynamic[6,`j'])
}

estat aggregation, dynamic(-5/10) ///
    graph(title("Dynamic ATET: never-treated controls") ///
    xtitle("Years since SSB tax started") ///
    ytitle("ATET on vol_pc_litres") ///
    name(dynamic_never, replace))
graph export "${PROJECT}/output/Figure1_dynamic_ATET_never.png", ///
    replace width(1800)
```

```
graph save "${PROJECT}/output/Figure1_dynamic_ATET_never.gph", replace

* 3.2 Robustness: regression adjustment and not-yet-treated controls.
use ` analysis_sample', clear
xtset cid Year

xthdidregress ra ///
    (vol_pc_litres ln_pop ln_gdp_ppp inflation) ///
    (tax_treat), ///
    group(cid) controlgroup(notyet) ///
    cohortvar(cohort_year_notyet, replace) nolog
estimates store did_ra_notyet
estimates save "${PROJECT}/output/did_ra_notyet.ster", replace

estat ptrends
post ` ptpost' ("Multi-period DID: not yet treated") ///
    (r(chi2)) (r(df)) (r(p))

estat aggregation, overall
matrix M_notyet = r(table)
post ` basepost' ("Robustness: not-yet-treated") ///
    (M_notyet[1,1]) (M_notyet[2,1]) (M_notyet[3,1]) ///
    (M_notyet[4,1]) (M_notyet[5,1]) (M_notyet[6,1]) ///
    (e(N)) (e(N_clust))

* 3.3 Auxiliary conventional two-way fixed-effects model.
use ` analysis_sample', clear
xtset cid Year
```

```
xtreg vol_pc_litres tax_treat ln_pop ln_gdp_ppp inflation i.Year, ///
    fe vce(cluster cid)
estimates store twfe_aux
estimates save "${PROJECT}/output/twfe_auxiliary.ster", replace

scalar twfe_b  = _b[tax_treat]
scalar twfe_se = _se[tax_treat]
scalar twfe_t  = twfe_b / twfe_se
scalar twfe_p  = 2 * ttail(e(df_r), abs(twfe_t))
scalar twfe_cv = invttail(e(df_r), 0.025)
scalar twfe_ll = twfe_b - twfe_cv * twfe_se
scalar twfe_ul = twfe_b + twfe_cv * twfe_se

post `basepost' ("Conventional TWFE") ///
    (twfe_b) (twfe_se) (twfe_t) (twfe_p) (twfe_ll) (twfe_ul) ///
    (e(N)) (e(N_clust))

postclose `basepost'
postclose `ptpost'
postclose `dynpost'

preserve
    use `baseline_results', clear
    format estimate se statistic p ll ul %12.3f
    export delimited using "${PROJECT}/output/Table2_baseline_results.csv", replace
    list, noobs abbreviate(30)
restore

preserve
```

```
    use `ptrends_results', clear
    format chi2 p %12.4f
    export delimited using "${PROJECT}/output/Parallel_trends_joint_tests.csv", replace
    list, noobs abbreviate(30)
restore

preserve
    use `dynamic_results', clear
    format atet se z p ll ul %12.3f
    export delimited using "${PROJECT}/output/Figure1_dynamic_ATET_estimates.csv", replace
    list, noobs
restore

******************************************************************************
* 4. TABLE 3: HETEROGENEITY BY HEALTH OBJECTIVE
*   Conventional TWFE interaction model among tax-adopting countries.
*
*   Y_it = alpha_i + lambda_t + beta1 Tax_it
*         + beta2 (Tax_it x Health_i) + gamma X_it + epsilon_it
*
*   beta1:       effect of non-health-objective taxes
*   beta2:       additional effect of an explicit health objective
*   beta1 + beta2: total effect of health-objective taxes
******************************************************************************

use `analysis_sample', clear
keep if ever_taxed
keep if !missing(health_obj_active)
```

```
* Health_i is time invariant within country and is absorbed by country FE.
bysort cid: egen byte health_obj_country = max(health_obj_active)
label variable health_obj_country "Tax has an explicit health objective"

* Audit whether each adopter has usable pre- and post-tax observations.
* The final reported specification retains all 32 eligible tax adopters and
* 476 complete observations. In the supplied data, 31 countries contribute an
* observed within-country treatment change; one adopter has no usable post-tax
* complete-case observation and therefore does not identify the treatment
* coefficient directly. Retaining it reproduces the final reported sample and
* estimates exactly.
bysort cid: egen byte has_pre_tax = max(tax_treat == 0)
bysort cid: egen byte has_post_tax = max(tax_treat == 1)
tabulate has_pre_tax has_post_tax if Year == 2010, missing

gen byte health_tax_active = tax_treat * health_obj_country
label variable health_tax_active "Active tax x health objective"

xtset cid Year

egen byte health_country_tag = tag(cid)
quietly count if health_country_tag
assert r(N) == 32
quietly count if health_country_tag & health_obj_country == 1
assert r(N) == 23
quietly count if health_country_tag & health_obj_country == 0
assert r(N) == 9
drop health_country_tag
assert _N == 476
```

```
xtreg vol_pc_litres tax_treat health_tax_active ///
    ln_pop ln_gdp_ppp inflation i.Year, fe vce(cluster cid)
estimates store twfe_health_objective
estimates save "${PROJECT}/output/twfe_health_objective.ster", replace

tempfile health_results health_sample
tempname healthpost healthcountpost
postfile ` healthpost' str42 item str20 expression double estimate se statistic p ll ul ///
    using ` health_results', replace
postfile ` healthcountpost' str28 group double countries observations ///
    using ` health_sample', replace

scalar h_cv = invttail(e(df_r), 0.025)

scalar h_b1  = _b[tax_treat]
scalar h_se1 = _se[tax_treat]
scalar h_t1  = h_b1 / h_se1
scalar h_p1  = 2 * ttail(e(df_r), abs(h_t1))
scalar h_ll1 = h_b1 - h_cv * h_se1
scalar h_ul1 = h_b1 + h_cv * h_se1
post ` healthpost' ("Effect of non-health-objective taxes") ("beta_1") ///
    (h_b1) (h_se1) (h_t1) (h_p1) (h_ll1) (h_ul1)

scalar h_b2  = _b[health_tax_active]
scalar h_se2 = _se[health_tax_active]
scalar h_t2  = h_b2 / h_se2
scalar h_p2  = 2 * ttail(e(df_r), abs(h_t2))
scalar h_ll2 = h_b2 - h_cv * h_se2
```

```
scalar h_ul2 = h_b2 + h_cv * h_se2
post `healthpost' ("Additional effect of health objective") ("beta_2") ///
    (h_b2) (h_se2) (h_t2) (h_p2) (h_ll2) (h_ul2)

lincom tax_treat + health_tax_active
scalar h_bt  = r(estimate)
scalar h_set = r(se)
scalar h_tt  = h_bt / h_set
scalar h_pt  = r(p)
scalar h_llt = r(lb)
scalar h_ult = r(ub)
post `healthpost' ("Total effect of health-objective taxes") ("beta_1 + beta_2") ///
    (h_bt) (h_set) (h_tt) (h_pt) (h_llt) (h_ult)

test health_tax_active = 0
display as result "Additional health-objective effect: F = " %9.3f r(F) ///
    ", p = " %9.4f r(p)

postclose `healthpost'

egen byte health_country_tag = tag(cid)
foreach h in 0 1 {
    local hlabel "Non-health-objective tax"
    if `h' == 1 local hlabel "Health-objective tax"
    quietly count if health_country_tag & health_obj_country == `h'
    local ncountries = r(N)
    quietly count if health_obj_country == `h'
    post `healthcountpost' ("`hlabel'") (`ncountries') (r(N))
}
```

```
quietly count if health_country_tag
local nhealthall = r(N)
quietly count
post ` healthcountpost' ("All tax-adopting countries") (` nhealthall') (r(N))
postclose ` healthcountpost'
drop health_country_tag

preserve
   use ` health_results', clear
   format estimate se statistic p ll ul %12.3f
   export delimited using "${PROJECT}/output/Table3_health_objective_TWFE.csv", replace
   list, noobs abbreviate(32)
restore

preserve
   use ` health_sample', clear
   export delimited using "${PROJECT}/output/Table3_health_objective_sample.csv", replace
   list, noobs
restore

********************************************************************************
* 5. TABLE 4: HETEROGENEITY BY TAX STRUCTURE
*    Each multi-period DID compares one tax type with never-treated countries.
********************************************************************************

use ` analysis_sample', clear

gen str12 tax_structure_country = strtrim(tax_structure) if ever_taxed
replace tax_structure_country = "" if !ever_taxed
```

```
label variable tax_structure_country "Country-level SSB tax structure"

* A tax-adopting country is used as treated only if at least one post-tax
* complete-case observation is available in the final estimation panel.
bysort cid: egen byte observed_treated_country = max(tax_treat)

tempfile tax_results tax_ptrends tax_twfe_results tax_tests
tempname taxpost taxptpost taxtwfepost taxtestpost

postfile `taxpost' str14 tax_structure double treated_countries N clusters ///
  atet se z p ll ul using `tax_results', replace
postfile `taxptpost' str14 tax_structure double chi2 df p ///
  using `tax_ptrends', replace

foreach t in "Ad valorem" "Mixed" "Specific" {
  local suffix = lower(strtoname("`t'"))

  preserve
    keep if !ever_taxed | ///
      (tax_structure_country == "`t'" & observed_treated_country == 1)
    xtset cid Year

    quietly levelsof cid if tax_structure_country == "`t'" & ///
      observed_treated_country == 1, local(treated_cids)
    local treated_count : word count `treated_cids'

    xthdidregress ra ///
      (vol_pc_litres ln_pop ln_gdp_ppp inflation) ///
      (tax_treat), ///
```

```
        group(cid) controlgroup(never) ///
        cohortvar(cohort_`suffix', replace) nolog
      estimates store did_tax_`suffix'
      estimates save "${PROJECT}/output/did_tax_`suffix'.ster", replace

      estat ptrends
      post `taxptpost' ("`t'") (r(chi2)) (r(df)) (r(p))

      estat aggregation, overall
      matrix M_tax = r(table)
      post `taxpost' ("`t'") (`treated_count') (e(N)) (e(N_clust)) ///
        (M_tax[1,1]) (M_tax[2,1]) (M_tax[3,1]) ///
        (M_tax[4,1]) (M_tax[5,1]) (M_tax[6,1])
    restore
}

postclose `taxpost'
postclose `taxptpost'

preserve
  use `tax_results', clear
  format atet se z p ll ul %12.3f
  export delimited using "${PROJECT}/output/Table4_tax_structure_MPDID.csv", replace
  list, noobs abbreviate(24)
restore

preserve
  use `tax_ptrends', clear
  format chi2 p %12.4f
```

```
    export delimited using "${PROJECT}/output/Table4_tax_structure_ptrends.csv", replace
    list, noobs
restore

******************************************************************************
* 6. AUXILIARY TAX-STRUCTURE TWFE AND BETWEEN-GROUP TESTS
******************************************************************************

gen byte tax_advalorem = tax_treat * (tax_structure_country == "Ad valorem")
gen byte tax_mixed     = tax_treat * (tax_structure_country == "Mixed")
gen byte tax_specific  = tax_treat * (tax_structure_country == "Specific")

label variable tax_advalorem "Ad valorem SSB tax in force"
label variable tax_mixed "Mixed SSB tax in force"
label variable tax_specific "Specific SSB tax in force"

xtset cid Year
xtreg vol_pc_litres tax_advalorem tax_mixed tax_specific ///
    ln_pop ln_gdp_ppp inflation i.Year, fe vce(cluster cid)
estimates store twfe_tax_structure
estimates save "${PROJECT}/output/twfe_tax_structure.ster", replace

postfile ` taxtwfepost' str14 tax_structure double estimate se t p ll ul ///
    using ` tax_twfe_results', replace

scalar ts_cv = invttail(e(df_r), 0.025)
foreach pair in "tax_advalorem|Ad valorem" "tax_mixed|Mixed" ///
    "tax_specific|Specific" {
    gettoken v label : pair, parse("|")
```

```
    gettoken pipe label : label, parse("|")

    scalar ts_b  = _b[`v']
    scalar ts_se = _se[`v']
    scalar ts_t  = ts_b / ts_se
    scalar ts_p  = 2 * ttail(e(df_r), abs(ts_t))
    scalar ts_ll = ts_b - ts_cv * ts_se
    scalar ts_ul = ts_b + ts_cv * ts_se
    post `taxtwfepost' ("`label'") (ts_b) (ts_se) (ts_t) (ts_p) (ts_ll) (ts_ul)
}
postclose `taxtwfepost'
postfile `taxtestpost' str34 comparison double F df_num df_den p ///
    using `tax_tests', replace
test tax_advalorem = tax_mixed
post `taxtestpost' ("Ad valorem = Mixed") (r(F)) (r(df)) (r(df_r)) (r(p))
test tax_advalorem = tax_specific
post `taxtestpost' ("Ad valorem = Specific") (r(F)) (r(df)) (r(df_r)) (r(p))
test tax_mixed = tax_specific
post `taxtestpost' ("Mixed = Specific") (r(F)) (r(df)) (r(df_r)) (r(p))
test tax_advalorem tax_mixed tax_specific
post `taxtestpost' ("Joint test: all tax types = 0") ///
    (r(F)) (r(df)) (r(df_r)) (r(p))
postclose `taxtestpost'
preserve
    use `tax_twfe_results', clear
    format estimate se t p ll ul %12.3f
    export delimited using "${PROJECT}/output/Tax_structure_auxiliary_TWFE.csv", replace
    list, noobs
restore
```

```
preserve
    use `tax_tests', clear
    format F p %12.4f
    export delimited using "${PROJECT}/output/Tax_structure_between_group_tests.csv", replace
    list, noobs abbreviate(32)
restore
*******************************************************************************
* 7. COMPLETION
*******************************************************************************
display as result "Reproduction completed successfully."
display as result "All tables, figures, estimates, and the log are in:"
display as result "${PROJECT}/output"
log close
```

5. Empirical Results:

Below is the Empirical Analysis of the Effect of Sugar-Sweetened Beverage Taxes on Per-capita soft-drink sales volume

5.1 Data Sources and Sample Construction

We use a geography-year dataset on soft-drinks sales and tax regulations. The primary data file is data.dta The original sample is a balanced panel of 96 geographies in the period 2010-2025. The total number of geography-year observations is 1,536. The country variable is Geography, the year variable is Year, the first year of SSB tax implementation is tax_start_year and the dependent variable is vol_pc_litres which is per capita soft drink consumption in litres. Note that the variable Category is Total Soft Drinks for all data.

Data construction follows the logic of cross-geography soft drink tax evaluation of Leibinger et al. (2025). The study analyses annual sales and composition data from Euromonitor International and shows the need of annual geography-level soft drink data for policy evaluation. It also observes that soft drink taxes are typically disclosed one to two years before to their formal introduction, thus policy discussion and announcement may trigger anticipatory responses from firms and consumers. This study builds a multi-period difference-in-differences framework based on the premise that soft drink consumption, tax timing and macroeconomic controls at the geography level.

Geographies with an SSB tax already in place at the beginning of the sample period in 2010 are dropped. These geographies were already pre-treated at the start of the panel, and as such, their pre-treatment consumption trends are not observable within the sample frame. Therefore, they cannot be used as newly treated units in a multi-period DID design. Excluded geographies include Argentina, Belgium, Brazil, Cambodia, Costa Rica, Croatia, El Salvador, Ethiopia, Finland, Guatemala, Laos, Latvia, Netherlands, Pakistan, Panama, Paraguay, Peru, Turkey and Uruguay. The maintained sample includes geographies that implemented an SSB tax after 2010 and geographies that did not enact an SSB tax.

Control variables include total population, GDP in current international dollars based on purchasing power parity and yearly consumer price inflation rate. Population and GDP are logged , i.e., ln(Population_total) and ln(GDP_PPP_current_intl_usd), to decrease scale-induced fluctuation. The final estimation sample includes 74 geographies and 1,096 geography-year data between 2010 and 2024 after removing geographies taxed by the baseline year and observations with missing outcome or control variables. Of these observations, 852 are untreated observations and 244 are observations after tax.

Table S5 Descriptive Statistics

Variable	Sample	Obs.	Mean	Std. dev.	Min.	Max.
Per capita soft drink consumption (vol_pc_litres)	Untreated	852	71.885	38.694	2.98	205.813

Per capita soft drink consumption (vol_pc_litres)	Post-tax	244	62.527	47.203	5.204	209.789
Log population (ln_pop)	Untreated	852	16.707	1.457	14.089	21.069
Log population (ln_pop)	Post-tax	244	17.351	1.249	14.734	21.095
Log GDP PPP (ln_gdp_ppp)	Untreated	852	26.786	1.453	23.874	31.274
Log GDP PPP (ln_gdp_ppp)	Post-tax	244	27.129	1.234	24.721	30.415
Inflation rate (inflation)	Untreated	852	4.816	12.197	-3.749	221.342
Inflation rate (inflation)	Post-tax	244	5.391	6.693	-2.54	49.721

The post-tax period refers to geography-years in which Year >= tax_start_year.

TABLE S6 FINAL ANALYTICAL SAMPLE OF 74 GEOGRAPHIES BY SSB TAX IMPLEMENTATION YEAR, TREATMENT STATUS, AND TAX STRUCTURE

Final analytical sample: 74 geographies				
**Geography**	**Tax implementation year**	**Treatment status in final analysis**	**Tax structure**	**Brief note**
Algeria	No tax	Untreated	No national tax	No national SSB tax during the study period
Angola	No tax	Untreated	No national tax	No national SSB tax during the study period
Australia	No tax	Untreated	No national tax	No national SSB tax during the study period
Austria	No tax	Untreated	No national tax	No national SSB tax during the study period
Bangladesh	2012	Treated	Ad valorem	Post-tax observations included in the final analysis
Belarus	No tax	Untreated	No national tax	No national SSB tax during the study period
Bolivia	2016	Treated	Specific	Post-tax observations included in the final analysis

Bosnia and Herzegovina	No tax	Untreated	No national tax	No national SSB tax during the study period
Bulgaria	No tax	Untreated	No national tax	No national SSB tax during the study period
Cameroon	2019	Treated	Mixed	Post-tax observations included in the final analysis
Canada	No tax	Untreated	No national tax	No national SSB tax during the study period
Chile	2014	Treated	Ad valorem	Post-tax observations included in the final analysis
China	No tax	Untreated	No national tax	No national SSB tax during the study period
Colombia	No tax	Untreated	No national tax	No national SSB tax during the study period
Czech Republic	No tax	Untreated	No national tax	No national SSB tax during the study period
Denmark	No tax	Untreated	No national tax	No national SSB tax during the study period
Dominican Republic	No tax	Untreated	No national tax	No national SSB tax during the study period
Ecuador	2016	Treated	Mixed	Post-tax observations included in the final analysis
Egypt	2016	Treated	Ad valorem	Post-tax observations included in the final analysis
Estonia	No tax	Untreated	No national tax	No national SSB tax during the study period

France	2012	Treated	Specific	Post-tax observations included in the final analysis
Georgia	No tax	Untreated	No national tax	No national SSB tax during the study period
Germany	No tax	Untreated	No national tax	No national SSB tax during the study period
Ghana	2014	Treated	Ad valorem	Post-tax observations included in the final analysis
Greece	No tax	Untreated	No national tax	No national SSB tax during the study period
Honduras	2020	Treated	Specific	Post-tax observations included in the final analysis
Hong Kong, China	No tax	Untreated	No national tax	No national SSB tax during the study period
Hungary	2011	Treated	Specific	Post-tax observations included in the final analysis
India	2017	Treated	Ad valorem	Post-tax observations included in the final analysis
Indonesia	No tax	Untreated	No national tax	No national SSB tax during the study period
Iraq	No tax	Untreated	No national tax	No national SSB tax during the study period
Ireland	2018	Treated	Specific	Post-tax observations included in the final analysis

Israel	No tax	Untreated	No national tax	No national SSB tax during the study period
Italy	No tax	Untreated	No national tax	No national SSB tax during the study period
Japan	No tax	Untreated	No national tax	No national SSB tax during the study period
Jordan	No tax	Untreated	No national tax	No national SSB tax during the study period
Kazakhstan	No tax	Untreated	No national tax	No national SSB tax during the study period
Kenya	2015	Treated	Specific	Post-tax observations included in the final analysis
Kuwait	No tax	Untreated	No national tax	No national SSB tax during the study period
Lebanon	No tax	Untreated	No national tax	No national SSB tax during the study period
Lithuania	No tax	Untreated	No national tax	No national SSB tax during the study period
Malaysia	2019	Treated	Specific	Post-tax observations included in the final analysis
Mexico	2014	Treated	Mixed	Post-tax observations included in the final analysis
Morocco	2019	Treated	Specific	Post-tax observations included in the final analysis
Myanmar	No tax	Untreated	No national tax	No national SSB tax during the study period

New Zealand	No tax	Untreated	No national tax	No national SSB tax during the study period
Nigeria	2021	Treated	Specific	Post-tax observations included in the final analysis
North Macedonia	No tax	Untreated	No national tax	No national SSB tax during the study period
Norway	No tax	Untreated	No national tax	No national SSB tax during the study period
Oman	2019	Treated	Ad valorem	Post-tax observations included in the final analysis
Philippines	2018	Treated	Specific	Post-tax observations included in the final analysis
Poland	2021	Treated	Specific	Post-tax observations included in the final analysis
Portugal	2017	Treated	Specific	Post-tax observations included in the final analysis
Qatar	2019	Treated	Ad valorem	Post-tax observations included in the final analysis
Romania	2023	Treated	Ad valorem	Post-tax observations included in the final analysis

Russia	2023	Untreated	Specific	Tax year identified, but no post-tax observations were available in the final complete-case sample
Saudi Arabia	2017	Treated	Ad valorem	Post-tax observations included in the final analysis
Serbia	No tax	Untreated	No national tax	No national SSB tax during the study period
Singapore	No tax	Untreated	No national tax	No national SSB tax during the study period
Slovakia	No tax	Untreated	No national tax	No national SSB tax during the study period
Slovenia	No tax	Untreated	No national tax	No national SSB tax during the study period
South Korea	No tax	Untreated	No national tax	No national SSB tax during the study period
Spain	2021	Treated	Ad valorem	Post-tax observations included in the final analysis
Sri Lanka	2018	Treated	Specific	Post-tax observations included in the final analysis
Sweden	No tax	Untreated	No national tax	No national SSB tax during the study period
Switzerland	No tax	Untreated	No national tax	No national SSB tax during the study period

Thailand	2017	Treated	Mixed	Post-tax observations included in the final analysis
Tunisia	2018	Treated	Ad valorem	Post-tax observations included in the final analysis
USA	No National tax	Untreated	No national tax	No national SSB tax during the study period
Uganda	2014	Treated	Mixed	Post-tax observations included in the final analysis
Ukraine	No tax	Untreated	No national tax	No national SSB tax during the study period
United Arab Emirates	2017	Treated	Ad valorem	Post-tax observations included in the final analysis
United Kingdom	2018	Treated	Specific	Post-tax observations included in the final analysis
Uzbekistan	No tax	Untreated	No national tax	No national SSB tax during the study period

5.2 Empirical Specification

SSB taxes were implemented in different geographies at different times. This setting is a typical staggered policy adoption design. Conventional two-way fixed effects models may produce biased weights when treatment effects are heterogeneous. Therefore, the main analysis uses the panel heterogeneous DID estimator implemented by Stata 18's xthdidregress command. The baseline model uses regression adjustment (RA), takes never-treated geographies as the control group, and controls for population, GDP, and inflation in the outcome model.

The treatment variable is constructed from each geography's first tax-start year:

Tax_it = 1(Year_t >= tax_start_year_i)

where Tax_it indicates whether geography i has implemented an SSB tax in year t. The main parameter of interest is the average treatment effect on the treated (ATET), which measures the

average difference between the observed per capita soft drink consumption of treated geographies after taxation and their counterfactual outcome without taxation:

ATET = E[Y_it(1) - Y_it(0) | Tax_it = 1]

Dynamic effects are further aggregated by the number of years relative to the tax-start year:

Exposure = Year - tax_start_year

Exposure = 0 denotes the implementation year, Exposure = 1 denotes the first year after implementation, and Exposure = -1 denotes the year before implementation. Standard errors are clustered at the geography level. As a robustness check, the paper also uses not-yet-treated geographies as the control group, namely controlgroup(notyet). In addition, a conventional two-way fixed effects (TWFE) model is reported as an auxiliary comparison. The TWFE results should be interpreted only as supporting evidence because, under staggered adoption and heterogeneous treatment effects, TWFE estimates may be affected by inappropriate comparisons and negative weights.

5.3 Baseline Regression Results

Table S7 Effect of SSB Taxes on Per Capita Soft Drink Consumption shows the overall treatment effect of SSB taxes on per capita soft drink consumption. In the baseline model, which includes never-treated geographies as the control group, SSB taxation reduces per capita soft drink consumption in the treated geographies by 7.286 litres on average and the effect is statistically significant at the 1% level. To test the robustness of the result to the definition of the control group, I choose not-yet-treated geographies as the control group. The estimated coefficient is -7.157 and still significant at the 1% level. The usual TWFE estimate is -5.140 (significant at 5% level), which has the same negative direction as the multi-period DID estimations.

Table S7 Effect of SSB Taxes on Per Capita Soft Drink Consumption

Variable	Baseline: never-treated controls	Robustness: not-yet-treated controls	Conventional TWFE
SSB tax (tax_treat)	-7.286***	-7.157***	-5.140**
	(2.21)	(2.17)	(2.45)
Controls	Yes	Yes	Yes
Year fixed effects	Handled by method	Handled by method	Yes
geography fixed effects	Handled by method	Handled by method	Yes
geography-clustered robust SE	Yes	Yes	Yes
Observations	1,096	1,096	1,096
geographies	74	74	74

Geography-clustered robust standard errors are reported in parentheses. ***, **, and * denote significance at the 1%, 5%, and 10% levels, respectively. The baseline and robustness models are estimated by xthdidregress ra. TWFE is reported as an auxiliary result.

In terms of economic scale, the average per capita soft drink consumption in the final sample is 69.801 litres. Hence, the baseline estimate of 7.286 litres is approximately 10.4% of the sample mean. This implies that SSB taxes are linked to economically relevant decreases in soft drink consumption compared to the counterfactual path.

This finding is consistent with the evidence. Andreyeva and colleagues' (2022) comprehensive review and meta-analysis of imposed SSB taxes found that SSB taxes were related with higher prices and reduced sales of taxed beverages. They find an average tax pass-through of roughly 82% and an average decline in SSB sales of about 15%. Silver et al. (2017) also find that the Berkeley SSB tax led to a decrease in the sales of taxed beverages and an increase in the sales of water and other untaxed beverages, suggesting a shift towards healthier beverages.

5.4 Dynamic Effects and Policy Anticipation

Figure below shows the dynamic ATET with never-treated geographies as the control group. The horizontal axis represents event time relative to the first year of SSB tax implementation. Event time 0 is the tax-start year, negative values are years before treatment and positive values are years after treatment. Overall, the ATET turns negative with the application of the tax and the extent of the negative effect increases over several years post-treatment. The ATET is -2.936 in the year of implementation, -4.221 in the first year after treatment, -5.651 in the second year after treatment and between about -9.664 and -11.056 litres from the fourth to the seventh year after treatment. The estimates for years 8 to 10 are still negative but the confidence ranges are broader and the statistical significance decreases, perhaps because fewer treated cohorts are available to assess longer term impacts.

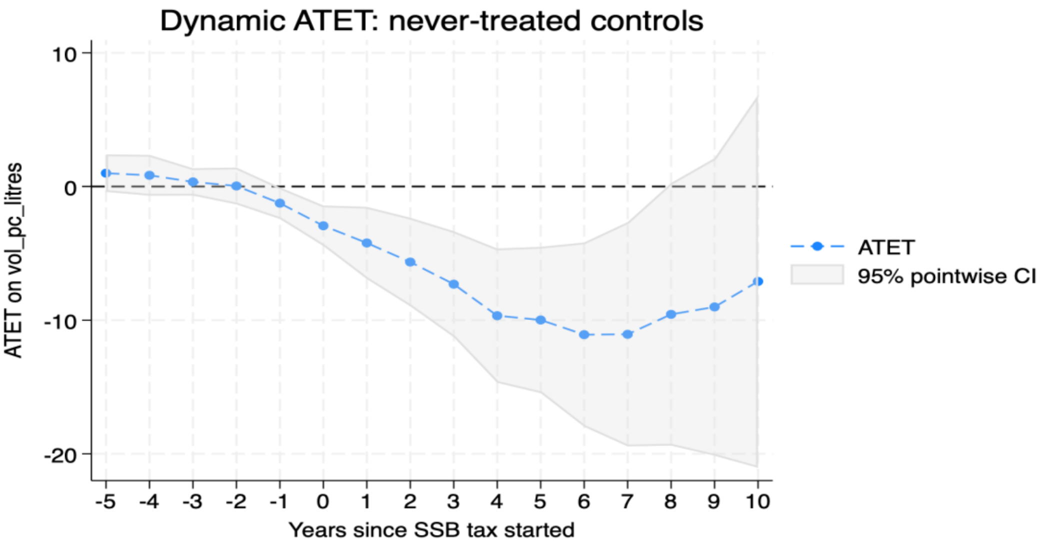

FIGURE S2 DYNAMIC ATET

Dynamic ATET: never-treated controls

The ATT from five to two years before implementation is close to zero and statistically insignificant, suggesting that no lasting meaningful pre-treatment difference may be found during the extended pre-policy window. However, the ATET one year before implementation is -1.247 and significant at 5%. This outcome can be viewed in terms of policy anticipation and transmission of health information. SSB taxes are not typically publicly disclosed on the formal tax start date. Instead, they are often the subject of legislative discussions, policy pronouncements, and media scrutiny before they are implemented. Leibinger et al. (2025) expressly states that soft drink tax schemes normally are publicised one to two years before formal implementation and that enterprises and market actors may begin to react prior to the policy being effective. An SSB tax also sends a message to consumers through legislation, media reporting, and public health communication that limiting sugar intake is good for health, in addition to changing relative pricing. This could lead to a consumption adjustment even prior to the formal imposition of the tax.

Theory and empirical facts also support this interpretation. According to Allcott et al. (2019), the case for SSB taxation is not only based on externalities but also on internalities, which are due to deficient nutrition information and self-control issues. Taxes may therefore affect consumption through both the price and information channels. The WHO guideline on fiscal policies for healthy diets also says that price changes can affect the demand for foods and beverages and that a major purpose of fiscal policy is to impact consumer behaviour. Berkeley data further indicate that when the SSB tax was enacted, consumers switched purchases from SSBs to water and other untaxed beverages. Thus, this article considers the pre-policy fall in the year prior to implementation as an anticipatory health reaction to policy discussion and projected taxation, rather than a mechanical price effect that occurs only after official implementation.

5.5 Heterogeneity by Health Objective

In this section, we investigate heterogeneity by policy purpose, using a common two-way fixed effects (TWFE) interaction model. This component differs from the baseline multi-period DID analysis in that we do not treat health-objective taxation as a separate multi-period DID treatment. It instead confines the sample to geographies that passed an SSB tax, and for which there exist pre- and post-policy observations. The model accounts for the overall tax-treatment variable and its interaction with the geography-level health-objective indicator. This specification disentangles the effect of non-health-objective taxes, the additional effect due to health-objective taxes, and the total effect of health-objective taxes.

The model is specified as follows:

$$Y\_it = \alpha\_i + \lambda\_t + \beta 1 \text{ Tax\_it} + \beta 2 \text{ Tax\_it} \times \text{Health\_i} + \varepsilon\_it$$

where Y_it is per capita soft drink consumption in geography i and year t. Tax_it indicates whether geography i has implemented an SSB tax in year t. Health_i indicates whether the geography's SSB tax has health as an explicit objective, and HealthTax_it = Tax_it x Health_i. X_it includes log population, log GDP PPP, and inflation. alpha_i and lambda_t denote geography fixed effects and year fixed effects, respectively. Standard errors are clustered at the geography level. Because

Health_i is time-invariant at the geography level, it is absorbed by the geography fixed effects and is not reported separately.

In this model, beta_1 evaluates the average effect of non-health-objective SSB tax. beta_2 captures the incremental impact of health-objective taxes above non-health-objective taxes. The sum beta_1+beta_2 is the overall effect of health-objective SSB taxes. Thus, a substantially negative beta_2 would imply that, in geographies with SSB taxes, a policy design with a clear health purpose has a more pronounced effect on decreasing consumption.

The TWFE interaction data are presented in Table S8 below. The sample comprises 32 tax adopting geographies and 476 geography-year observations (23 health objective tax geographies and 9 non-health objective tax geographies). The coefficient of non-health-objective taxes is -1.036 but is not statistically significant. The additional effect of health-objective taxes is -5.814 and is significant at the 5% level, which shows that health-objective taxes further lower per capita soft drink consumption compared to non-health-objective taxes. The outcome of the linear combination shows that the total effect of the health-objective taxes is -6.850 and significant at the 1% level. These results are consistent with the notion that the impact of SSB taxation is not just determined by taxes but also by whether the policy has a declared public health purpose.

Table S8 Health-Objective Heterogeneity: TWFE Interaction Model

Item	Statistical expression	Coefficient	Std. error	p-value	Interpretation
Effect of non-health-objective taxes	beta_1	-1.036	1.628	0.529	Average effect of non-health-objective SSB taxes; not significant
Additional effect of health objective	beta_2	-5.814**	2.671	0.037	Additional decline relative to non-health-objective taxes
Total effect of health-objective taxes	beta_1 + beta_2	-6.850***	2.218	0.004	Overall effect after health-objective tax implementation

The model accounts for geography fixed effects, year fixed effects, log population, log GDP PPP and inflation. Standard errors are clustered at the geography level. The F-statistic for the test of the additional impact H0: beta_2 = 0 is 4.74 and p = 0.0372. ***, **, * refer to significance at the level of 1%, 5% and 10% correspondingly.

5.6 Heterogeneity by Tax Structure

The variable tax_structure captures the type of SSB tax in each geography. The tax structures that may be identified in the baseline sample are ad valorem taxes, mixed taxes and specific taxes. We keep geographies with each tax type and never-treated geographies separately, using the same sample structure and control-variable specification as in the baseline model, and estimate the ATET of each tax type relative to never-treated controls using xthdidregress ra. If no post-tax observations are observed for a geography in the current complete sample, it is not counted as a treated geography for the specific tax type.

Table S9 below presents the multiperiod DID results per tax structure. The calculated coefficients on ad valorem and mixed taxes are -9.890 and -10.747 respectively, both significant at the 5% level or greater. This means that these two tax regimes are associated with bigger reductions in per capita soft drink consumption. The coefficient of specific taxes is -3.437, slightly significant at the 10 percent threshold. Its sign is also negative but the statistical significance and the economic magnitude are smaller than the other two tax systems.

Table S9 Tax-Structure Heterogeneity: Multi-Period DID Results

Tax structure	Treated countries	Obs.	Clusters	ATET	Std. error	p-value	95% CI
Ad valorem	12	799	54	-9.890***	3.733	0.008	[-17.206, -2.574]
Mixed	5	695	47	-10.747**	4.614	0.02	[-19.790, -1.704]
Specific	14	830	56	-3.437*	2.024	0.089	[-7.404, 0.529]

Each row is estimated via a different multi-period DID regression. The treatment group is the respective tax-structure group and the control group is the never treated group. Controls: ln_pop, ln_gdp_ppp, inflation ***, ** and * signify significance at 1%, 5% and 10% levels respectively.

To further evaluate variations across tax structures, the article additionally builds three policy interaction variables, tax_advalorem, tax_mixed, and tax_specific, and includes them simultaneously in a nation and year fixed effects model. All coefficients for ad valorem taxes, mixed taxes and specific taxes are negative, but the between-group difference tests are not statistically significant: the p-value for the difference between ad valorem and mixed taxes is 0.883, the p-value for the difference between ad valorem and specific taxes is 0.440 and the p-value for the difference between mixed and specific taxes is 0.672. Thus, the results of the heterogeneity analysis should be understood as point estimates for ad valorem and mixed taxes being higher and more obvious, not statistically significant differences between tax forms.

References

Andreyeva, T., Marple, K., Marinello, S., Moore, T. E., & Powell, L. M. (2022). Outcomes following taxation of sugar-sweetened beverages: A systematic review and meta-analysis. JAMA Network Open. https://jamanetwork.com/journals/jamanetworkopen/fullarticle/2792842

Allcott, H., Lockwood, B. B., & Taubinsky, D. (2019). Should we tax sugar-sweetened beverages? An overview of theory and evidence. Journal of Economic Perspectives. https://www.aeaweb.org/articles?id=10.1257/jep.33.3.202

Leibinger, A., et al. (2025). The impact of tiered soft drink taxes in Europe on mean sales-weighted sugar content of soft drinks: A quasi-experimental study. BMC Public Health. https://link.springer.com/article/10.1186/s12889-025-23331-w

Silver, L. D., Ng, S. W., Ryan-Ibarra, S., Taillie, L. S., Induni, M., Miles, D. R., Poti, J. M., & Popkin, B. M. (2017). Changes in prices, sales, consumer spending, and beverage consumption one year after a tax on sugar-sweetened beverages in Berkeley, California, US: A before-and-after study. PLOS Medicine. https://journals.plos.org/plosmedicine/article?id=10.1371/journal.pmed.1002283

World Health Organization. (2024). Fiscal policies to promote healthy diets: WHO guideline. https://www.who.int/publications/i/item/9789240091016

Euromonitor International. Passport: soft drinks [database]. London: Euromonitor International; 2024.

World Bank. Global Sugar-Sweetened Beverage Tax Database: August 2023 version [Internet]. Washington, DC: World Bank; 2023. Available from: https://ssbtax.worldbank.org/

World Bank. World Development Indicators [Internet]. Washington, DC: World Bank; 2025. Available from: https://data.worldbank.org/
